# Educational Case: Platelet refractoriness

**DOI:** 10.1016/j.acpath.2022.100015

**Published:** 2022-05-12

**Authors:** Alex J. Griffith, William N. Rose

**Affiliations:** Department of Pathology, University of Wisconsin Hospital, Madison, WI, USA

**Keywords:** Pathology competencies, Organ system pathology, Hematopathology—Platelets and coagulation disorders, Thrombocytopenia, Platelet refractoriness

The following fictional case is intended as a learning tool within the Pathology Competencies forMedical Education (PCME), a set of national standards for teaching pathology. These are divided into three basic competencies: Disease Mechanisms and Processes, Organ System Pathology, and Diagnostic Medicine and Therapeutic Pathology. For additional information, and a full list of learning objectives for all three competencies, see https://www.journals.elsevier.com/academic-pathology/news/pathology-competencies-for-medical-education-pcme.^1^

## Primary objective

Objective HPCD1.2: Thrombocytopenia. Identify the examples of each of the following pathogenetic categories of thrombocytopenia: decreased production, decreased platelet survival, sequestration, dilutional effect.

Competency 2: Organ system pathology; Topic: Hematopathology—platelets and coagulation disorders (HPCD); Learning goal 1: Platelets.

## Patient presentation

A hospitalized 65-year-old woman with transfusion dependence secondary to bone marrow ablative chemotherapy for treatment of acute myeloid leukemia (AML) was evaluated by the transfusion medicine service due to concern for platelet refractoriness. After initiating induction therapy one month ago, the patient developed persistent thrombocytopenia and now requires one platelet transfusion per day with the aim of maintaining an adequate platelet count until the patient is able to receive a bone marrow graft. In the past three months, she has received 37 units of apheresis platelets as well as 13 units of RBCs.

The medicine resident has a complete blood count drawn at around 6 a.m. The platelet count is assessed with a goal of > 10,000 ​cells/μL. However, despite repeated platelet transfusions, the platelet count remains below this threshold. She reports no complaints other than fatigue. Daily physical exam has been unremarkable with no lymphadenopathy, splenomegaly, hepatomegaly, petechiae, purpura, or bruising. She has remained hemodynamically stable with vital signs within normal limits.

## Diagnostic findings, Part 1

Results of the complete blood count are presented in Table 1. The platelet counts for the three days prior have all been < 10,000 ​cells/μL with selected historical results depicted in Table 2.

**Table 1:** Complete blood count.

Laboratory test	Result	Reference interval
White blood cell count	3.7	4.0–10.4 (cells x 10^3^/μL)
Red blood cell count	3.4	4.36–5.78 (cells x 10^6^/μL)
Hemoglobin	9.4	13.8–17.3 (g/dL)
Hematocrit	30	39.5–50.2 (%)
Mean corpuscular volume	88.2	81-95 (fL)
Mean corpuscular hemoglobin concentration	31.3	32.8–36.4 (g/dL)
Mean corpuscular hemoglobin	27.6	27–35 ​pg
Red cell distribution width	13.9	< 14.2 (%)
Platelet count	5	141-377 (cells x 10^3^/μL)

**Table 2:** Platelet counts.

Day of hospitalization	Platelet count result (cells x 10^3^/μL)
Day 1	5
Day 2	5
Day 3	5
Day 4	5

## Questions/discussion points, Part 1

### How would you interpret the patient's complete blood count in Table 1?

The patient is anemic, leukopenia, and thrombocytopenic. With a low hemoglobin (along with hematocrit and RBC count) and MCV within normal limits, the patient has a normocytic anemia. Normocytic anemia has a broad differential diagnosis but considering this patient's history, her anemia is most likely secondary to her bone marrow ablative chemotherapy with her chronic disease contributing as well. Similarly, the patient's leukopenia is most likely due to bone marrow suppression. Her thrombocytopenia will be focused on for the remainder of this article.

### What are the potential causes of thrombocytopenia in a patient undergoing treatment for acute leukemia?

In a patient receiving chemotherapy for treatment of acute leukemia, the most likely cause of thrombocytopenia is bone marrow suppression. Other causes of decreased production of platelets may also be considered, such as aplastic anemia or a bone marrow infiltrative process.

Thrombocytopenia may also occur due to increased destruction of platelets. Etiologies within this category include immune disorders (e.g. idiopathic thrombocytopenic purpura and drug induced thrombocytopenia), sepsis, liver failure, viral infections, and preeclampsia/eclampsia. Based on the lack of splenomegaly, splenic sequestration is not likely in this patient. Direct loss via bleeding is also unlikely.

Another possible cause of thrombocytopenia in this case would be dilution, especially considering the patient's transfusion dependence. Dilution effect is often seen after significant blood loss (approximately 15–25% of the total blood volume) and/or excess intravenous fluids such as saline. However, the blood loss in this patient is not at the usual magnitude of blood loss that is consistent with the dilution effect.

### Define platelet refractoriness

Platelet refractoriness refers to the failure to achieve an adequate response to a platelet transfusion from a random donor on at least two consecutive transfusions. Whether a response is “adequate” can be determined clinically (for example, cessation of bleeding) and/or by using a variety of formulae such as the corrected count increment (CCI) which will be discussed in a later section.

### What are the causes of platelet refractoriness?

The causes of platelet refractoriness include immune and non-immune etiologies.^2^ Table 3 lists common causes from both categories. Immune causes may be due to human leukocyte antigen, human platelet antigen, and/or ABO alloantibodies. Other antibodies such as drug-induced antibodies and plasma protein antibodies may also be involved. One important consideration is that the presence of alloimmunization does not necessarily mean clinical immune refractoriness will be present.^3^ Non-immune causes of platelet refractoriness have a broad range of etiologies and are more common than immune causes.^4^

**Table 3:** Platelet refractoriness etiology.

Immune Causes	Non-immune causes
HLA alloimmunization	Fever
HPAalloimmunization	Sepsis
ABO incompatibility	Splenomegaly
Drug-induced antibodies	Disseminated intravascular coagulation
Plasma protein antibodies	Bleeding
	Veno-occlusive disease
	Graft-versus-host disease
	Mechanical platelet destruction (e.g., artificial heart valve)

## Diagnostic findings, Part 2

The blood bank pathologist on call is consulted and recommends that platelet counts be drawn within 10–60 ​min after platelet transfusion. He states that a CCI will be calculated after each transfusion and will be used to direct next steps. The hematology resident manages to obtain a platelet count during these post-transfusion intervals. The following two platelet counts and CCIs are presented in Table 4.

**Table 4:** Platelet counts and corrected count increments (CCIs).

Pre-transfusion platelet count(x 103/μL)	Post-transfusion platelet count(x 103/μL)	Time after platelet transfusion/	CCI^a^
5	10	50 ​min	2.5 ​× ​109/L (or 2500/μL)
5	10	46 ​min	2.5 ​× ​109/L (or 2500/μL)

a The patient's body surface area was 2 ​m^2^ and there were 4.0 ​× ​1011 platelets transfused in each apheresis unit.

## Questions/discussion points, Part 2

### What is the importance of obtaining platelet counts within 10–60 ​min of platelet transfusion?

Obtaining a platelet count during this interval can help determine the broad etiology of refractoriness.^5^ Clinical trials that assess post-transfusion counts often use a 1-h post-transfusion count. Therefore, obtaining timely lab work allows for standardization and comparison based on the available literature.

Also, because alloimmunization tends to affect platelets more quickly than non-immune causes, waiting several hours to draw the specimen can sometimes make it impossible to distinguish between immune and non-immune etiologies. Patients that have a non-immune cause of refractoriness usually will have a normal transfusion response 10–60 ​min post-transfusion, while patients with an immune cause will typically show marginal or no increase in platelet count.^6^

### What formulae are used to assess for platelet refractoriness?

The post-transfusion platelet increment (PPI) is the difference in platelet count between pre- and post-transfusion.

Because patients vary in size and platelet units vary in dose, the CCI controls for body surface area (BSA) and the dose used for transfusion. The CCI is the PPI times the BSA divided by the dose of platelets.

The units of the CCI are usually listed in one of two ways: as a number/uL or a number x 10^9^/L. This case will use the format of a number x 10^9^/L.

One example calculation is the following. If the patient has a PPI of 30k/uL, BSA of 2 ​m^2^, and dose of 4 ​× ​10^11^, then the CCI = (PPI)x(BSA)/dose ​= ​30 ​× ​2/4 ​= ​15 ​× ​10^9^/L.

A CCI <5 on two consecutive occasions suggests immune refractoriness. A CCI>10 is considered a good transfusion response. CCIs between 5 and 10 may be considered a gray area. Some authors designate 7.5 as the sole threshold without a gray area.

## Diagnostic findings, Part 3

In this case, the CCI was used to assess the effectiveness of the transfusion. The CCI for the patient's first and second transfusion are shown in Table 4.

Two consecutive platelet transfusions demonstrated 1-hCCIs of less than five. The blood bank pathologist suspects an immune component for the patient's platelet refractoriness. A platelet antibody screen is recommended, and the screen is positive.

Platelet crossmatching is then performed, and crossmatch-compatible units are identified and set aside for this patient.

## Questions/discussion points, Part 3

### What caused the immune refractoriness in this patient?

Based on her initial history, the most likely explanation is that she has developed one or more antibodies to HLAs as the result of several transfusions of platelets and RBCs, both of which contain some WBCs.

HLA alloimmunization is also commonly caused by pregnancy. The patient's pregnancy history is not known, but it may have contributed in addition to the dozens of transfusions.

### How can antibodies that cause platelet refractoriness be detected?

If the 1-h CCI is less than five for at least two consecutive platelet transfusions, then an immune cause of platelet refractoriness should be investigated. Two options for detection of relevant antibodies include class I HLA antibody testing and platelet antibody testing. If HLA antibody testing is performed and yields a sufficiently positive result, then the patient may be administered HLA-matched platelets. Alternatively, if a platelet antibody screen is performed and yields a positive result, then the patient may be administered crossmatched platelets.

### What are HLA-matched platelets? What are the pros and cons?

The first step toward providing the patient with HLA-matched platelets is ordering HLA antibody testing. This may be done along with HLA class I phenotyping if it has not already been performed. HLA-matching consists of pairing the patient with platelet samples that are either well-matched (based on the HLA class I phenotyping) and/or lack cognate antigens (based on antibody testing). Of note, platelets express HLA class I antigens but not class II.

Compared to crossmatching, HLA-matching is more precise and predictable. Specific antibodies and antigens are detected. Also, this method helps to prevent the formation of additional antibodies by matching the transfused platelets as close as possible to the patient's HLA phenotype.

However, HLA-matching is not as widely available because not every hospital has an HLA lab. HLA-matching is also usually more expensive and often has a longer turnaround time compared to crossmatched platelets. Even if an on-site HLA lab is present, the tests may be batched and delayed by a few days. In addition, there is a risk of wasted time due to the relatively long turnaround time and typical additional step of finding donors who can donate on demand.

### What are crossmatched platelets? What are the pros and cons?

The first step toward providing the patient with crossmatched platelets is performing a platelet antibody screen. This involves mixing the patient plasma with reagent platelets with known class I HLA and human platelet antigens (HPA). In this scenario antibodies to HLA antigens are a more likely cause compared to HPA antigens. Indicator red blood cells coated with anti-human globin are then added. If antibodies in the patient's plasma bind with reagent platelet antigens, the indicator anti-human globulin coated red blood cells will bind to the Fc portion of the patient's antibodies. This red blood cell adherence creates a visible endpoint that is considered positive. Testing is considered negative if there is no red blood cell adherence in mixing the patient's plasma with any of the reagent platelets used. In this case no further testing is needed.

If one or more reagent platelets react with patient plasma, then platelet crossmatching is the next step. This involves a similar process to the platelet antibody screen described above, except instead of using reagent platelets, platelets from the current blood bank inventory are used. The samples that have the least reactivity with patient plasma are usually issued preferentially.

Of note, while this platelet crossmatching is being performed, the shelf platelets used must be quarantined. This is to ensure that the most compatible units will be available when testing is complete.

Two advantages of crossmatching are speed and often a higher likelihood finding a compatible donor. A disadvantage is that repeated crossmatching may be required if the patient has prolonged immune refractoriness, as a platelet unit's shelf life is five days.

### What are important steps after administering HLA-matched platelets or crossmatched platelets?

It is important to continue to assess platelet counts 10–60 ​min after transfusions in order to calculate CCIs. This allows for continued assessment of transfusion response and for determination if platelet products from certain donors yield satisfactory results. If so, those donors may be asked to donate again. An implication of this is that *matching does not guarantee clinical benefit*.

### What role does ABO compatibility play in platelet refractoriness?

Platelets have ABH antigens on their membranes. If a patient has isoagglutinins to the donor's platelet antigens, this may decrease the efficacy of the transfusion. ABO incompatibility is often not clinically significant in the setting of a platelet transfusion, but ensuring ABO compatibility can be done in some cases to increase the platelet increment slightly.^7^

In clinical practice there may arise situations in which a patient requires a timely platelet transfusion, but HLA or crossmatched platelets are unavailable. In such scenarios clinicians may consider providing ABO-identical or ABO major compatible platelets—evaluating compatibility in an analogous fashion to how red blood cell ABO compatibility is determined.

### What is the clinical management of non-immune platelet refractoriness?

While an exhaustive discussion is beyond our scope, the management of non-immune causes of platelet refractoriness typically involves addressing the underlying cause(s). This may involve treating an underlying infection, stopping offending medications, or identifying, and stopping sources of bleeding.

### What was the outcome of this patient?

The platelet crossmatch was able to identify several crossmatch-compatible units. Subsequent transfusions using the crossmatched platelets resulted in a CCI greater than five for most but not all of the matched products. Later in the hospital stay, the patient underwent a bone marrow transplant. The transplant engrafted, and the patient's platelet counts are now consistently in the normal range.

## Teaching points

• Thrombocytopenia can be caused by one or more of the following etiologies: decreased production (e.g. due to a marrow disorder such as acute leukemia), decreased survival (e.g. destruction due to allo- or autoantibodies), sequestration (e.g. due to splenomegaly), or dilution (e.g. after significant blood loss and/or excess saline administration).
• Platelet refractoriness refers to the failure to achieve an adequate response to a platelet transfusion from at least two consecutive random donor transfusions.
• Two categories of causes of platelet refractoriness are immune and non-immune etiologies. Non-immune causes are more common that immune. Both may be present.
• The corrected count increment (CCI) 10–60 ​min after transfusion is the first step in assessing the etiology(ies).
• If the CCI is less than 5 for two consecutive platelet transfusions, then an immune cause of platelet refractoriness should be investigated.
• Two options for detection of relevant antibodies include class I HLA antibody testing and platelet antibody testing.
• If HLA antibody testing is performed and yields a sufficiently positive result, then the patient may be administered HLA-matched platelets. Similarly, if a platelet antibody screen is performed and yields a positive result, then the patient may be administered crossmatched platelets.
• After each HLA-matched or crossmatched platelet unit, it is important to continue to assess platelet counts 10–60 ​min after transfusions in order to calculate CCIs.
• Non-immune refractoriness may be managed by addressing the underlying causes.
• A positive antibody test, by itself, does *not* diagnose refractoriness.
• Low CCIs alone do *not* diagnose immune refractoriness and do *not* indicate special products.
• Both low CCIs and a positive antibody test do *not* guarantee a matched product will yield a benefit.
• An underappreciated nuance is that the optimal assessment of platelet refractoriness is *step-wise.* The important steps are the following and in this sequence. Obtain post-transfusion counts within 60 ​min of transfusion. Calculate CCI. If 2 consecutive CCIs are low, then do antibody testing. If antibody testing is sufficiently positive, procure matched platelets. After each transfusion of matched platelets, obtain a post-transfusion count within 60 ​min and calculate CCI. If the CCI is good, then inform the blood center to consider asking the donor to donate again for the patient.
